# Characteristics and associated factors of acute kidney injury among adult dengue patients: A retrospective single-center study

**DOI:** 10.1371/journal.pone.0210360

**Published:** 2019-01-07

**Authors:** Ajib Diptyanusa, Weerapong Phumratanaprapin, Benjaluck Phonrat, Kittiyod Poovorawan, Borimas Hanboonkunupakarn, Natthida Sriboonvorakul, Usa Thisyakorn

**Affiliations:** 1 Department of Clinical Tropical Medicine, Faculty of Tropical Medicine, Mahidol University, Bangkok, Thailand; 2 Department of Parasitology, Faculty of Medicine, Public Health and Nursing, Universitas Gadjah Mada, Yogyakarta, Indonesia; University of Sao Paulo Medical School, BRAZIL

## Abstract

Severe dengue cases have been increasingly reported in Thailand, and the under-reporting of acute kidney injury (AKI) in cases of dengue viral infection has become an obstacle in obtaining an accurate description of the true nature and epidemiology of AKI. Because AKI may lead to patient morbidity and mortality, an early diagnosis is important in preventing its onset in dengue patients. This study aimed to determine the prevalence, clinical and laboratory characteristics, and associated factors of AKI among adult dengue patients. This retrospective study reviewed admission data from the medical records of adult dengue patients admitted to the Bangkok Hospital for Tropical Diseases between January 2012 and November 2017 and stratified these patients into AKI and non-AKI groups using the Kidney Disease Improving Global Outcomes criteria (KDIGO). A total of 1,484 patients were included in the study, with 71 categorized into the AKI group. The prevalence of AKI was 4.8%. In the AKI group, the predominant age range was 18–40 years (71.8%), with a female to male ratio of 1:2.7. These patients showed significantly (*P* < 0.05) higher proportions of altered consciousness, dyspnea, low mean arterial blood pressure, high-grade fever, major bleeding, severe thrombocytopenia, hypoalbuminemia, severe transaminitis, coagulopathy, metabolic acidosis, rhabdomyolysis, proteinuria, hematuria, and pyuria. Our study established that older age, male sex, diabetes mellitus, obesity, severe dengue, and coexisting bacterial infection were significant associated factors for AKI in dengue by multivariate analysis. A total of 10 (14.1%) patients with AKI received dialysis, among which 9 (12.7%) patients from the AKI group died. Our findings suggest that an awareness of AKI, its early diagnosis, and evaluation of clinical and laboratory characteristics of dengue patients will help clinicians to initiate appropriate therapy for dengue-associated AKI.

## Introduction

Dengue is one of the most important and fastest growing, mosquito-borne viral diseases affecting tropical and subtropical regions in the world, particularly the Asia Pacific region [1]. Approximately two-fifth of the world’s population, including an estimated 3.9 billion people in 128 countries, lives in areas at a high risk for dengue transmission [2]. The number of reported dengue cases has been increasing over the past 5 years, with recurring epidemics and an increasing proportion of severe dengue cases, particularly in Thailand, Indonesia, and Myanmar [3]. A recent report by the Ministry of Public Health of Thailand showed that a total of 31,392 dengue cases were identified in 77 provinces in 2017, with a morbidity rate of 47.98 per 100,000 people [4].

Acute kidney injury (AKI) is a disorder that affects people worldwide and has high morbidity and mortality. There is a large burden of AKI cases in Asian countries, many of which have been identified in the more tropical regions. The reported incidence of AKI in a hospital setting was 19.4% in Eastern Asia, 7.5% in Southern Asia, 9.0% in Central Asia, 16.7% in Western Asia, and 31.0% in Southeastern Asia [5]. In contrast to AKI in non-tropical regions, AKI in the tropics predominantly occurs in younger population and is mainly caused by infections. The incidence of AKI in tropical acute febrile illnesses is 41.1%; among 7.6% of patients with dengue, more than a third had AKI [6]. Several studies have reported that the prevalence of dengue-associated AKI ranges from 0.9% to as high as 69.4% [7–9]. Dengue is therefore considered to be one of the major causes of AKI in the tropics [6].

Although the exact mechanism of dengue virus infection (DVI) in AKI is unclear, several theories have been proposed, including direct injury [10], an indirect mechanism via the immune system [11], hemolysis or rhabdomyolysis [12], and hypotensive mechanisms resulting from shock [13]. The development of AKI continues to be associated with poor DVI outcomes. Additionally, AKI in DVI doubles the length of a typical hospital stay [14]. Dengue patients with AKI were reported to have case-fatality of 0.9%–60% [9, 15, 16]. However, a lack of multi-center studies and the under-reporting of AKI in the tropics are obstacles in understanding its true epidemiology. The high prevalence of dengue-associated AKI previously reported in Malaysia [15, 17] encourages more studies to be conducted, particularly in the developing countries of Southeast Asia. Until recently, there has been only one similar study conducted in children in Thailand [8]. Owing to the increasing number of severe dengue cases reported in Thailand over the past decade, further vigorous investigations should be undertaken to help clinicians make accurate early diagnoses to help prevent AKI. Therefore, the present study aimed to determine the prevalence, clinical and laboratory characteristics, and associated factors of AKI among adult dengue patients.

## Materials and methods

This retrospective study evaluated the medical records of adult dengue patients admitted to the Bangkok Hospital for Tropical Diseases, Faculty of Tropical Medicine, Mahidol University, Thailand between January 2012 and November 2017. Dengue patients were identified using the International Statistical Classification of Diseases and Related Health Problems, 10th Revision (ICD-10) for DVI [18]. Laboratory-confirmed dengue patients aged ≥18 years who were hospitalized for ≥2 days were included in the current study. This laboratory confirmation of DVI cases was performed using at least one of the following tests: (1) positive dengue nonstructural protein 1 (NS1) result, (2) presence of dengue IgM antibodies in acute phase serum by enzyme-linked immunosorbent assay (ELISA), (3) presence of dengue IgG antibodies in seroconversion serum by ELISA, or (4) positive polymerase chain reaction result. Patients with incomplete information regarding their demographics, physical examination findings, serum creatinine (SCr) levels, or complete blood count were excluded from the study. Eligible patients were reviewed and stratified into AKI and non-AKI groups using the Kidney Disease Improving Global Outcomes (KDIGO) criteria [19]. SCr was determined by using an enzymatic method.

Data from both hard copies and computerized records of each patient were retrieved to assemble patient demographics as well as clinical and laboratory findings, all of which were recorded using a structured case record form. Demographics and clinical presentations were noted at hospital admission, whereas laboratory data were followed-up throughout the period of hospitalization.

### AKI definition

AKI was determined using the KDIGO criteria as per this protocol: stage 1: SCr increase ≥ 0.3 mg/dL or SCr increase 1.5–1.9 times from baseline; stage 2: SCr increase 2–2.9 times from baseline; stage 3: SCr increase three times from baseline, or initiation of renal replacement therapy (RRT). The SCr data on admission were chosen as the baseline value for the determination of AKI. For patients with only a single SCr result, the baseline SCr was estimated using the Modification of Diet in Renal Disease (MDRD) equation by assuming a glomerular filtration rate (GFR) of 75 ml/min/1.73 m^2^ [20].

### General definitions

The World Health Organization (WHO) 1997 criteria was applied as the standard for the case definition of DVI, including dengue fever (DF), dengue hemorrhagic fever (DHF), and dengue shock syndrome (DSS) [21]. The WHO 2009 case definitions were also used for severe dengue [22].

An adult is defined as a person aged 18 years or older. Late hospital admission is defined as hospital admission on day 5 of illness or later. Low mean arterial pressure (MAP) is defined as MAP <65 mmHg, and obesity is defined as BMI >27.5 kg/m^2^, as adjusted for Asian population parameters.

Hyperbilirubinemia is defined as a total serum bilirubin concentration >1.2 mg/dL. Hypoalbuminemia is defined as serum albumin concentration <3.5 g/dL. Transaminitis (elevated aspartate aminotransferase (AST) or alanine aminotransferase (ALT), or both) is further classified into mild (elevation <3x the upper normal limit), moderate (elevation 3–10x the upper normal limit), severe (elevation >10x the upper normal limit). Coagulopathy is defined as prolonged PT (>13 s) or prolonged aPTT (>35 s).

We considered metabolic acidosis to be defined as a serum HCO_3_ <15 mmol/L. Likewise proteinuria is defined as protein urine ≥30 mg/dL or dipstick urine protein +1 or above; hematuria is defined as RBC ≥5 cells/HPF, or dipstick urine blood +2 or above; pyuria is defined as presence of WBC ≥3 cells/HPF, or a urine dipstick positive for leukocyte esterase. Rhabdomyolysis is defined as elevated creatine phosphokinase (CPK) >5x upper normal limit.

Severe thrombocytopenia is defined as thrombocyte count <50,000 cells/mm^3^. Shock is defined as circulatory failure (rapid and weak pulse, narrow pulse pressure ≤20 mmHg, or hypotension) with signs of reduced tissue perfusion. Respiratory failure is defined as respiratory rate >35 breaths/min, or use of mechanical ventilator. Coexisting bacterial infection is defined by isolation of a pathogen from culture, identification of a pathogen from a specimen staining technique, or positive serological assay for a specific pathogen. Encephalopathy is defined as an occurrence of seizure and altered consciousness during a period of illness. Myocarditis is defined as N-terminal pro b-type natriuretic peptide (NT pro-BNP) >450 ng/L, or creatine kinase-MB (CKMB) >25 U/L, or Troponin I >0.02 ng/mL [23]. Multi-organ failure is defined as presence of ≥2 organs failure.

### Ethics statement

This study received ethical approval (Certificate No. MUTM 2017-068-01) from the Ethics Committee of the Faculty of Tropical Medicine, Mahidol University. The Ethics Committee waived the requirement for informed consent and the data were fully anonymized before analysis.

### Statistical analysis

The sample size was calculated using a formula according to primary objective for prevalence estimation [24] based on previous study [7] for an error of 1%, at 95% confidence interval (CI). Sample size calculation was also adjusted to include secondary objective, which was to determine associated factors for AKI by using a formula for two independent proportions (two-tailed) [24], with alpha of 0.05% and power of 80%. Finally, a minimum sample size of 1,476 was sufficient for this study. Statistical analysis was conducted using SPSS version 18.0 (SPSS, IL, USA). Qualitative variables were expressed as frequencies along with percentages. Quantitative variables were presented as median and interquartile range (IQR) due to a skewed data distribution resulting even after transforming the data using logarithmic functions. The chi-square test, Fisher’s exact test, and Mann–Whitney tests were performed, wherever appropriate. Selected variables with a *P*-value <0.10 in univariate analysis were further analyzed using a stepwise multivariate logistic regression model to obtain independent associated factors of AKI [adjusted odds ratio (OR) with 95% CI]. Forward stepwise method was used with entry at 0.05 and removal at 0.10. All variables were classified to be statistically significant if their two-tailed *P*-value was <0.05.

## Results

**Fig 1:**
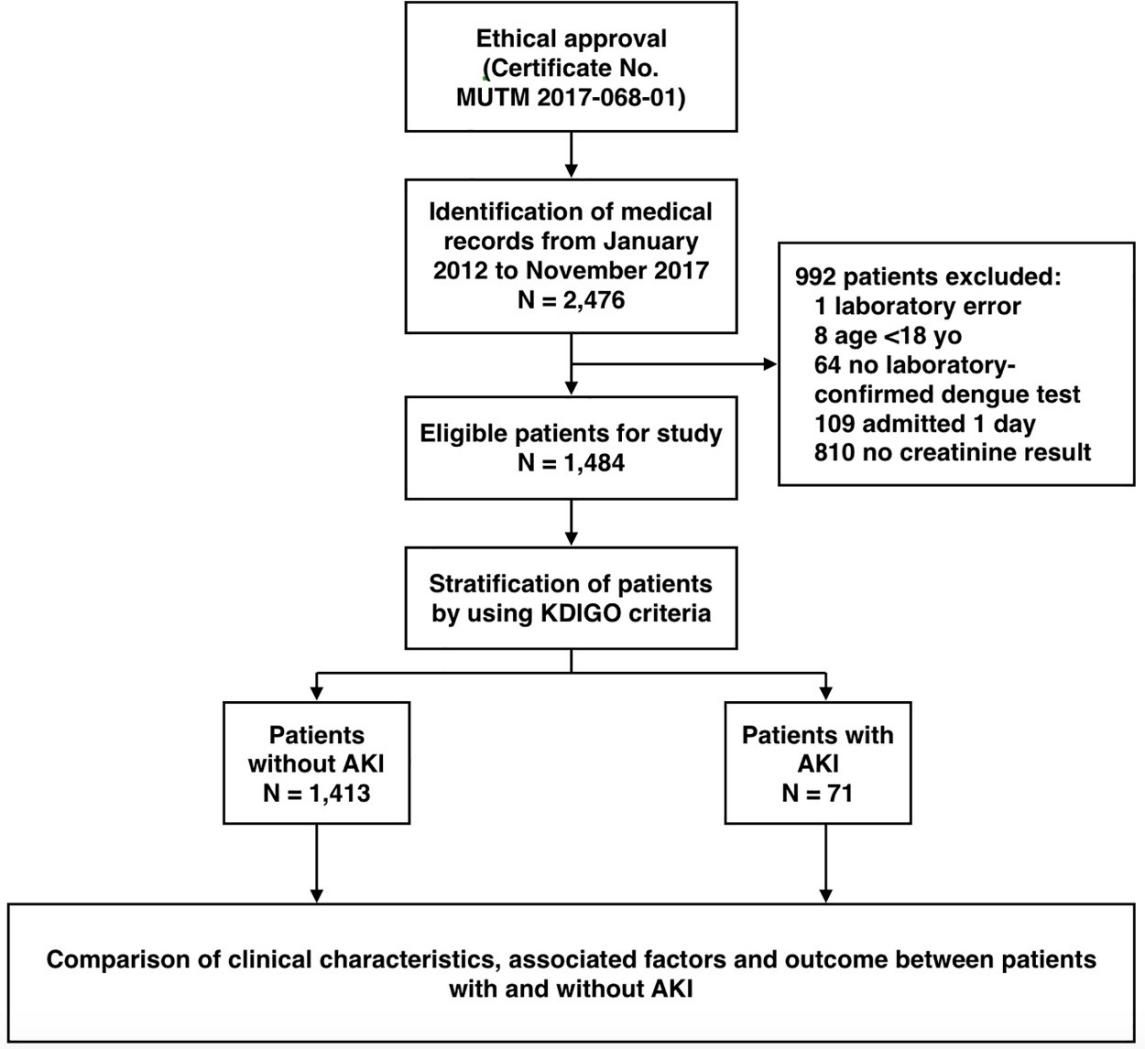
Study flow diagram.

A total of 2,476 medical records of patients coded with DVI were screened for enrollment in this study, of which 992 cases were excluded. Finally, 1,484 patients were enrolled and eligible for analysis, as shown in Fig 1. Approximately 60% (891 cases) of our total patients were estimated for baseline SCr using the MDRD formula.

### Characteristics of study patients

Totally, 1,413 patients were categorized into the non-AKI group, whereas the remaining 71 were categorized into the AKI group. The prevalence of AKI was 4.8%. Of these, 59 (83.1%) patients were classified as stage 1 AKI, 3 (4.2%) as stage 2 AKI, and 9 (12.7%) as stage 3 AKI. In contrast to the non-AKI group, the AKI group mostly comprised males (52/71, 71.8%; *P* < 0.001), with a female to male ratio of 1:2.7. The clinical characteristics of patients with DVI having AKI during hospitalization are shown in Table 1. In the AKI group, 25 (35.2%) patients were classified as DF and 46 (64.8%) as DHF (*P* < 0.001). All DHF grade IV cases were found in the AKI group. Using the WHO 2009 dengue case definition, approximately 29.6% (21/71) of patients in the AKI group were classified as severe (*P* < 0.001).

**Table 1:** Demographics and clinical characteristics among DVI patients with and without AKI.

Parameters	Total patients N = 1,484	Non-AKI N = 1,413	AKI N = 71	*P*-value
Age^†^	28 (22–40)	28 (22–40)	31 (24–43)	0.073^§^
Age group^‡^				
18–40 years	1,118 (75.3)	1,067 (75.5)	51 (71.8)	0.081**
41–60 years	286 (19.3)	274 (19.4)	12 (16.9)	
>60 years	80 (5.4)	72 (5.1)	8 (11.3)	
Sex^‡^				
Female	736 (49.6)	717 (50.7)	19 (26.8)	<<0.001**
Male	748 (50.4)	696 (49.3)	52 (73.2)	
BMI^†^	22.4 (19.7–25.8)	22.2 (19.7–25.7)	25.0 (22.3–29.5)	<0.001^§^
Pre-morbid diseases^‡^				
Diabetes mellitus	78 (5.3)	66 (4.7)	12 (16.9)	<0.001*
Hypertension	173 (11.7)	157 (11.1)	16 (22.5)	0.006**
CKD	8 (0.5)	3 (0.2)	5 (7.0)	<0.001*
Hepatitis B infection	26 (1.8)	15 (1.1)	11 (15.5)	<0.001*
Hematologic disorders^a^	45 (3.0)	38 (2.7)	7 (9.9)	0.004*
Other kidney diseases^b^	7 (0.5)	6 (0.4)	1 (1.4)	0.291*
HIV infection	9 (0.6)	9 (0.6)	0 (0)	N/A
Altered consciousness^‡^	12 (0.8)	1 (0.1)	11 (15.5)	<0.001*
Dyspnea^‡^	34 (2.3)	20 (1.4)	14 (19.7)	<0.001*
Nausea and/or vomiting^‡^ (N = 1,468)	672 (45.8)	639 (45.8)	33 (46.5)	0.999**
Low MAP^c^^‡^	37 (2.5)	22 (1.6)	15 (21.1)	<0.001*
High-grade fever^‡^	142 (9.6)	125 (8.8)	17 (23.9)	<<0.001**
Major bleeding^d^^‡^	27 (1.8)	22 (1.6)	5 (7.0)	0.008*
WHO 1997 classification^‡^				
DF	964 (65.0)	939 (66.5)	25 (35.2)	<<0.001**
DHF	520 (35.0)	474 (33.5)	46 (64.8)	
Grade I	195 (13.1)	185 (13.1)	10 (14.1)	
Grade II	308 (20.7)	283 (20.0)	25 (35.2)	
Grade III	7 (0.5)	6 (0.4)	1 (1.4)	
Grade IV	10 (0.7)	0 (0)	10 (14.1)	
WHO 2009 classification^‡^				
Non-severe dengue	1385 (93.3)	1335 (94.5)	50 (70.4)	<0.001*
Severe dengue	99 (6.7)	78 (5.5)	21 (29.6)	

CKD: chronic kidney disease; HIV: human immunodeficiency virus; MAP: mean arterial pressure

^a^hematologic disorders: G6PD deficiency, thalassemia, anemia, polycythemia vera, Idiopathic Thrombocytopenic Purpura (ITP)

^b^other kidney diseases: kidney stone, benign prostatic hyperplasia, polycystic kidney disease, IgA nephropathy

^c^low MAP: MAP <65 mmHg

^d^major bleeding: GI bleeding, hemothorax, hemoperitoneum, intracranial hemorrhage

^†^Median (IQR)

^‡^Frequency (%)

^§^Mann–Whitney *U* test

*Fisher’s exact test

**chi-square test

Laboratory parameters of patients recorded on admission are presented in Table 2. Statistically significant differences were observed in the medians of serum sodium, serum potassium, serum bicarbonate, serum total bilirubin, AST, ALT, urine specific gravity, WBC count, neutrophils, lymphocytes, atypical lymphocytes, PT, INR, aPTT, CPK, and serum lactate levels (*p* < 0.05). In the AKI group, 10 patients presented with serum creatinine levels of >3.0 mg/dL on admission. In addition, in the AKI group, 30.5% (18/59) of patients had hyperbilirubinemia during hospitalization, with a very high serum total bilirubin level of 27.6 mg/dL reported for one patient. Approximately 73.1% (49/67) of the AKI cases were categorized as having moderate to severe transaminitis, along with very high AST (>48,000 U/L) and ALT (>26,000 U/L) values observed during the hospitalization period, each found in different patients. The median urine specific gravity in the AKI group was 1.020 (1.013–1.025; *P* < 0.001). Urine sediment findings in the AKI group included proteinuria (38/54, 70.4%), hematuria (32/54, 59.3%), and pyuria (22/54, 40.7%), all of which showed statistical significance (*P* < 0.05).

**Table 2:** Laboratory characteristics among patients with DVI with and without AKI.

Parameters	Overall		Non-AKI		AKI		*P*-value^§^
	N	Median (IQR)	N	Median (IQR)	N	Median (IQR)	
WBC (×10^3^/μL)	1,484	3.3 (2.4–4.8)	1,413	3.3 (2.4–4.7)	71	4.5 (2.5–5.5)	0.013
Neutrophils (%)	1,484	48.0 (34.0–63.0)	1,413	48.0 (33.0–62.9)	71	55.0 (43.0–64.0)	0.009
Lymphocytes (%)	1,484	27.0 (19.0–37.0)	1,413	28.0 (19.0–37.0)	71	23.0 (14.0–30.0)	0.001
Atypical lymphocytes (%)	1,484	10.0 (5.0–18.0)	1,413	10.0 (5.0–18.0)	71	9.0 (4.0–13.0)	0.038
RBC (×10^6^/μL)	1,484	5.07 (4.60––5.55)	1,413	5.07 (4.61–5.54)	71	4.99 (4.41–5.75)	0.625
Platelet (×10^3^/μL)	1,484	67 (37–104)	1,413	68 (37–104)	71	63 (35–88)	0.141
Hemoglobin (g/dL)	1,484	13.9 (12.8–15.2)	1,413	14.0 (12.9–15.2)	71	13.8 (12.7–15.4)	0.671
Hematocrit (%)	1,484	41.2 (38.3–44.7)	1,413	41.3 (38.3–44.6)	71	40.7 (37.3–45.1)	0.648
Serum creatinine (mg/dL)^a^	1,484	0.80 (0.67–1.00)	1,413	0.80 (0.65–0.98)	71	1.23 (1.05–1.69)	N/A
BUN (mg/dL)	1,463	9.1 (6.9–11.9)	1,392	8.9 (6.8–11.6)	71	15.8 (11.1–18.8)	N/A
Serum Na (mmol/L)	1,387	136 (134–139)	1,317	137 (134–139)	70	135 (133–137)	<0.001
Serum K (mmol/L)	1,387	3.7 (3.4–3.9)	1,317	3.7 (3.4–3.9)	70	3.9 (3.6–4.2)	<0.001
Serum HCO_3_ (mmol/L)	1,387	24 (22–26)	1,317	24 (23–26)	70	23 (20–24)	<0.001
Serum albumin (g/L)	1,069	4.1 (3.7–4.3)	1,008	4.1 (3.7–4.3)	61	4.1 (3.5–4.3)	0.248
Serum total bilirubin (mg/dL)	1,051	0.4 (0.3–1.0)	992	0.4 (0.3–0.6)	59	0.6 (0.4–1.2)	<0.001
AST (IU/L)	1,415	101 (57–213)	1,348	99 (56–203)	67	188 (88–929)	<0.001
ALT (IU/L)	1,414	65 (33–142)	1,347	65 (33–138)	67	124 (46–406)	<0.001
PT (s)	371	11.7 (11.0–12.6)	329	11.6 (10.9–12.5)	42	12.6 (11.8–15.5)	<0.001
INR	371	1.02 (0.94–1.10)	329	1.01 (0.93–1.09)	42	1.11 (1.01–1.46)	<0.001
aPTT (s)	363	31.2 (28.5–35.3)	323	30.9 (28.3–34.2)	40	35.5 (30.6–43.5)	<0.001
CPK (U/L)	282	152 (82–296)	250	144 (79–265)	32	354 (173–762)	<0.001
Serum lactate (mg/dL)	239	13.0 (10.7–16.8)	205	12.8 (10.7–15.7)	34	18.5 (11.3–111.0)	<0.001

WBC: white blood cell; RBC: red blood cell; BUN: blood urea nitrogen; AST: aspartate aminotransferase; ALT: alanine aminotransferase; PT: prothrombin time; INR: international normalized ratio; aPTT: activated partial thromboplastin time; CPK: creatine phosphokinase

^a^normal reference range according to hospital values: 0.67–1.17 mg/dl for males and 0.51–0.95 mg/dl for females

^§^Mann–Whitney *U* test

### Patient complications and outcomes

Complications observed in the AKI group included: severe thrombocytopenia (53/71, 74.6%), hypoalbuminemia (32/61, 52.5%), severe transaminitis (27/67, 40.3%), coagulopathy (32/42, 76.2%), and rhabdomyolysis (5/32, 15.6%). All of these showed statistical significance (*P* < 0.05). Shock was observed in 11 (15.5%) patients; coexisting bacterial infection was observed in 15 (21.1%) patients; respiratory failure requiring mechanical ventilator was observed in 11 (15.5%) patients; hemodialysis was required in 10 (14.1%) patients; metabolic acidosis was observed in 17 (24.3%) patients; major bleeding was observed in 5 (7.0%) patients; encephalopathy was observed in 5 (7.0%) patients; myocarditis was observed in 2 (2.8%) patients; DIC was found in 7 (9.9%) patients; multi-organ failure was detected in 12 (16.9%) patients. Shock, coexisting bacterial infections, metabolic acidosis, major bleeding, encephalopathy, myocarditis, and multi-organ failure showed statistical significance (*P* < 0.05).

Vasopressors were administered to approximately 0.1% (2/1,413) of the patients in the non-AKI group and 12.7% (9/71) of patients in the AKI group, all of whom were in shock. Fortunately, all patients in the non-AKI group had completely recovered at the time of discharge, whereas death occurred in 9 (12.7%) patients from the AKI group (Table 3). All of these patients had both liver and respiratory failure and were also in shock during hospitalization. Furthermore, all of the death cases were categorized as DHF grade IV, severe dengue, and AKI stage 3.

**Table 3:** Complications and outcomes among patients with DVI with and without AKI.

Parameters^‡^	Overall patients N = 1,484	Non-AKI N = 1,413	AKI N = 71	*P*-value
**Complications**				
Severe thrombocytopenia	873 (58.8)	820 (58.0)	53 (74.6)	0.006**
Hypoalbuminemia (N = 1,069)	190 (17.8)	158 (15.7)	32 (52.5)	<0.001**
Severe transaminitis (N = 1,415)	231 (16.3)	204 (15.1)	27 (40.3)	<0.001**
Coagulopathy (N = 371)	164 (44.2)	132 (40.1)	32 (76.2)	<0.001**
Rhabdomyolysis (N = 282)	15 (5.3)	10 (4.0)	5 (15.6)	0.018*
Shock	17 (1.1)	6 (0.4)	11 (15.6)	<0.001*
Coexisting bacterial infection	51 (3.4)	36 (2.5)	15 (21.1)	<0.001*
Respiratory failure	11 (0.7)	0 (0)	11 (15.5)	N/A
Metabolic acidosis	41 (3.0)	24 (1.8)	17 (24.3)	<0.001*
Major bleeding	27 (1.8)	22 (1.6)	5 (7.0)	0.008*
Encephalopathy	6 (0.4)	1 (0.1)	5 (7.0)	<0.001*
Myocarditis	5 (0.3)	3 (0.2)	2 (2.8)	0.021*
DIC	7 (0.5)	0 (0)	7 (9.9)	N/A
Multi-organ failure	13 (0.9)	1 (0.1)	12 (16.9)	<0.001*
**Outcome**				
Dialysis	10 (0.7)	0 (0)	10 (14.1)	N/A
Hospitalization >3 days	671 (45.2)	627 (44.4)	44 (62.0)	0.005**
Use of vasopressors	11 (0.7)	2 (0.1)	9 (12.7)	<0.001*
Recovery	1,475 (99.4)	1,413 (100)	62 (87.3)	<0.001*
Death	9 (0.6)	0 (0)	9 (12.7)	N/A

DIC: disseminated intravascular coagulation

^‡^Frequency (%)

*Fisher’s exact test

**chi-square test

### Independent factors associated with AKI

Based on univariate analyses and a thorough literature review, the following independent variables were placed into the full model: age (adjusted by 10 years), male sex, history of diabetes mellitus, history of hepatitis B infection, obesity, hypoalbuminemia on admission, severe transaminitis on admission, severe dengue, and coexisting bacterial infection. Categorical variables were placed into the model with entry at 0.05 and removal at 0.10 and were scored using ‘no’ as the reference category. After placing these numerous variables into the model, two confounding variables were eliminated: hypoalbuminemia and severe transaminitis. Finally, the following variables were established to be significantly associated with AKI in our present study: age (adjusted by 10 years), male sex, diabetes mellitus, obesity, severe dengue, and coexisting bacterial infection.

From our analyses, the following variables were statistically likely to promote the development of AKI: an increase in 10 years of age (AOR 1.30; 95% CI, 1.03–1.64), male sex (AOR 3.49; 95% CI, 1.75–6.95), a history of diabetes mellitus (AOR 2.89; 95% CI, 1.05–7.93), obesity (AOR 1.94; 95% CI, 1.00–3.77), severe dengue (AOR 5.45; 95% CI, 2.47–12.04), and coexisting bacterial infection (AOR 6.15; 95% CI, 2.57–14.74). The current multivariate regression model demonstrated a sensitivity of 81.8% (95% CI, 48.2%–97.7%), specificity of 95.2% (95% CI, 93.6%–96.5%), positive predictive value of 16.9% (95% CI, 12.0%–23.4%), and negative predictive value of 99.8% (95% CI, 99.2%–99.9%). The results of univariate and multivariate analysis are shown in Table 4.

**Table 4:** Independent associated factors for AKI by univariate and multivariate analyses.

Variables	Univariate analysis		Multivariate analysis	
	*P*-value	COR (95% CI)	*P*-value	AOR (95% CI)
Age (adjusted by 10 years)	0.073^§^		0.027	1.30 (1.03–1.64)
Male sex	<0.001**	2.82 (1.65–4.81)	<0.001	3.49 (1.75–6.95)
Diabetes mellitus	<0.001*	4.15 (2.13–8.10)	0.039	2.89 (1.05–7.93)
Hepatitis B infection	<0.001*	17.09 (7.53–38.78)	0.054	3.46 (0.98–12.23)
Obesity	<0.001**	2.77 (1.61–4.76)	0.049	1.94 (1.00–3.77)
Hypoalbuminemia	0.003**	2.70 (1.43–5.08)	-	
Severe transaminitis	<0.001**	3.94 (2.35–6.71)	-	
Severe dengue	<0.001*	7.18 (4.11–12.56)	<0.001	5.45 (2.47–12.04)
Coexisting bacterial infection	<0.001*	10.24 (5.30–19.80)	<0.001	6.15 (2.57–14.74)

COR: crude odds ratio; AOR: adjusted odds ratio

^§^Mann–Whitney *U* test

*Fisher’s exact test

**chi-square test

**Fig 2:**
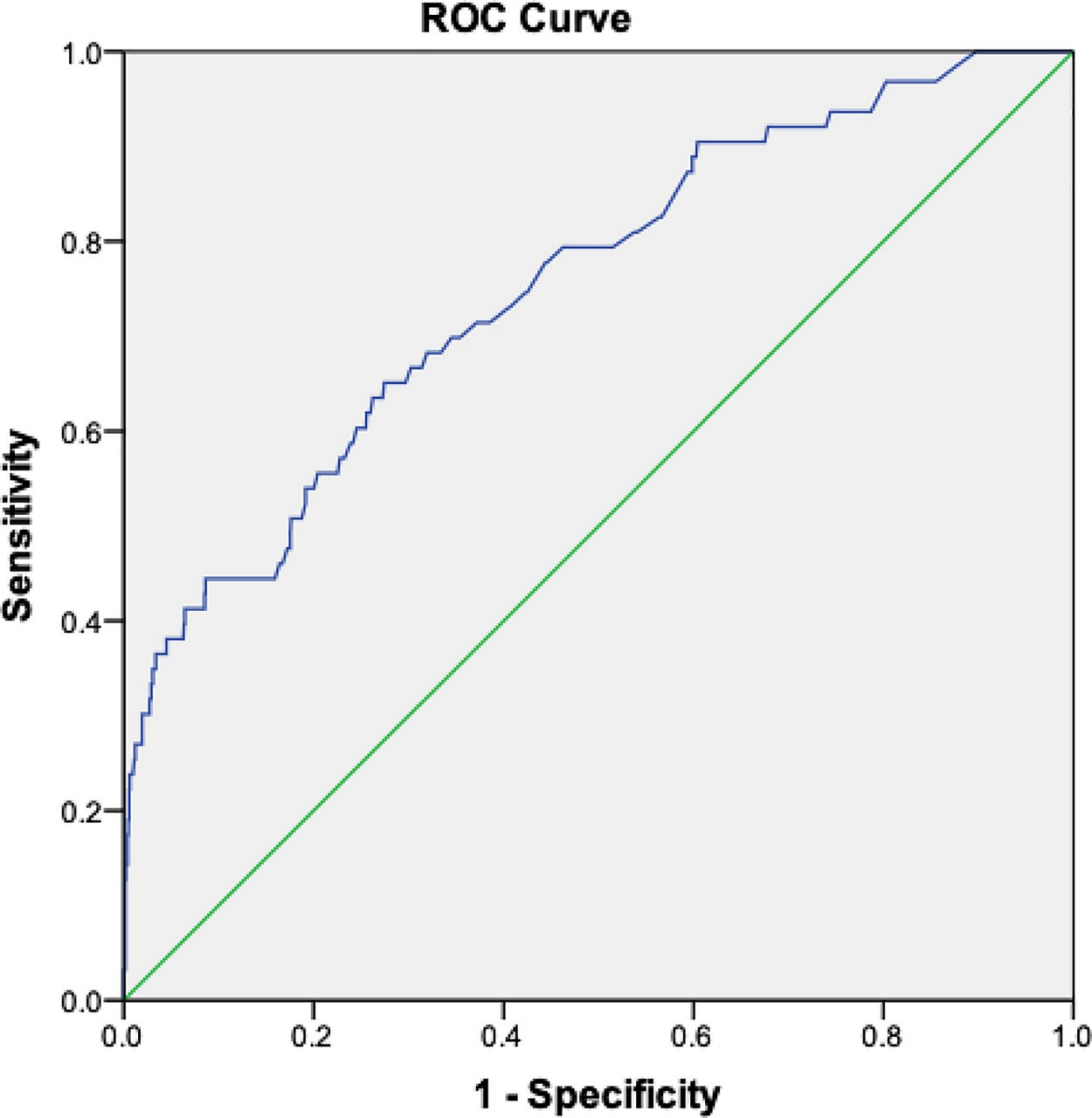
ROC curve analysis of multivariate regression model of AKI among dengue patients.

A receiver operating characteristic (ROC) curve was generated from the multivariate regression model, which yielded an area under the curve of 0.753 (95% CI, 0.687–0.819) (Fig 2). Based on maximum sensitivity and specificity for the model, optimum probability cut-off point of 0.0446 was selected, which yielded sensitivity of 64.5% and specificity of 72.6%.

## Discussion

This large-scale, single-center retrospective study assessed the association of AKI with DVI. Studies performed in Taiwan in 2008 [7] and in Malaysia in 2016 [25] reported an AKI prevalence of approximately 4.0%, which is slightly lower than that reported in our study (4.8%). Both studies were conducted using almost similar age groups, were performed in all DVI spectrums, and used the Risk, Injury, Failure, Loss, and End-stage kidney disease (RIFLE) criteria as the basic diagnostic criteria for AKI. In contrast, a study published in 2017 [15] and another in 2018 [17] using the Acute Kidney Injury Network (AKIN) criteria found much different AKI prevalence of 20.7% and 13.7%, respectively. A majority of patients included in these studies had severe dengue. An even higher AKI prevalence in adult dengue patients was reported in India in 2016 using the KDIGO criteria [9]: 69.4%, which may have been very high owing to a small sample size. However, that particular study did not mention the severity of DVI in the included patients. Several studies conducted among critically ill, non-dengue patients admitted to an intensive care unit (ICU) demonstrated that the KDIGO criteria were more sensitive than their RIFLE criteria and were either better or equal to AKIN criteria in detecting the presence of AKI in those populations [26, 27]. Another study also reported that the KDIGO criteria were more sensitive than the RIFLE and AKIN criteria in detecting AKI in non-ICU patients [28]. Nevertheless, the current study used admission SCr as baseline SCr that may have underestimated severity and prognosis of patients.

Unfortunately, our study excluded approximately 40% of the total screened medical records, and of these cases, 81.7% were due to the simple absence of a creatinine investigation. These patients might not have shown signs of AKI during hospitalization, and therefore, clinicians did not send these patients for creatinine analyses. Similarly, other studies excluded as low as 5% and up to 74% of patients due to the absence of creatinine results [7, 15].

The pathogenesis of AKI in DVI is complex, and therefore, it is difficult to determine the exact mechanism involved. Nevertheless, it is assumed that dengue-associated AKI results from either direct injury [10], an indirect mechanism via the immune system [11], hemolysis or rhabdomyolysis [12], or hypotensive mechanisms resulting from shock [13]. Histopathological analyses were not performed for our patients, and therefore, we could not demonstrate the evidence of direct injury or immune-mediated mechanisms. Direct invasion of the dengue virus and the production of cytotoxic cytokines may result in rhabdomyolysis, causing AKI [12]. In our study, less than 50% of the patients in the AKI group were investigated for CPK levels, the results of which may not represent an actual association despite being statistically significant (*P* = 0.018). A total of 16 (22.2%) patients in the AKI group had a MAP of <65 mmHg that is associated with shock. Shock-induced AKI may cause a reduced or even total loss of the renal autoregulation function and a low MAP. In our study, however, only 11 (15.3%) patients advanced into shock. Although the mechanism of AKI in dengue is unclear, the most commonly assumed cause includes hypotensive mechanisms resulting from shock [13].

High-grade fever was notable in 17 (23.6%) patients from the AKI group. The presence of a high-grade fever may further contribute to dehydration in dengue patients, in parallel with hypovolemia due to plasma leakage and bleeding. Almost all patients who developed altered levels of consciousness belonged to the AKI group (0.1% and 15.3% in non-AKI and AKI groups, respectively). Such altered consciousness in patients might have resulted from shock, electrolyte disturbances, or uremic encephalopathy. During the period of hospitalization, 14 (19.4%) patients in the AKI group developed dyspnea, among which 11 (78.6%) progressed into respiratory failure in need of mechanical ventilation. Dyspnea with concurrent metabolic acidosis, along with the presence of shock, are the most common conditions associated with AKI in dengue [10].

We observed statistically significant median differences in WBC count, along with its differential counts, for patients in the AKI group. In this context, neutrophils generate reactive oxygen species, leading to tubular cell damage, whereas lymphocytes play a role in the production of pro-inflammatory cytokines, which lead to the inflammatory process of AKI [29]. A high neutrophil–lymphocyte ratio has been reported to increase the risk of developing AKI [30]. Severe thrombocytopenia may also contribute to the bleeding tendency in dengue patients. Despite being very rare, dengue patients have been known to present with AKI due to thrombotic microangiopathy characterized by severe thrombocytopenia and hemolytic anemia. As for direct nephrotoxic drug use, only four patients had received tenofovir and four had taken NSAIDs during their illness, and none of them belonged to the AKI group. Therefore, we could not put this data into our statistical analysis.

Hypoalbuminemia has been proven to be a risk factor for the development of AKI. Yet, patients with AKI did not show statistically significant median differences in albumin levels compared with those without AKI in the current study. Severe transaminitis was observed in 40.3% (27/67) of the patients, whereas 30.5% (18/59) of those in the AKI group had hyperbilirubinemia. Dengue patients with elevated transaminases >1,000 U/L, particularly if coexisting with hyperbilirubinemia, hypoalbuminemia, and prolonged PT and aPTT, are more likely to develop AKI. The binding of the dengue virus onto hepatocytes will initiate hepatocellular apoptosis and later trigger the enhancement of a host’s immune reaction, causing a cytokine storm that results in elevated transaminase levels [31]. The increase in AST levels was more prominent than that in ALT levels, which suggests that cellular injury by dengue virus from extrahepatic sources contributes to AST elevation. Bilirubin may act as a direct nephrotoxin and may promote renal susceptibility to ischemia [32], which causes AKI, particularly if the patient presents with hyperbilirubinemia. Although coagulopathy is also a common risk factor, its exact mechanism in the pathogenesis of dengue-associated AKI remains unclear.

A decrease in GFR will result in retention of waste products, which in turn will lead to electrolyte disturbances, including hyponatremia, hyperkalemia, and metabolic acidosis. Our study showed statistically significant median differences in serum sodium, potassium, and bicarbonate levels between the AKI and non-AKI groups. Metabolic acidosis is commonly found in dengue-associated AKI. In the current study, patients in the AKI group showed a higher proportion of metabolic acidosis in comparison to those in the non-AKI group. However, complications of patients in current study may not be solely due to DVI itself, because pre-morbid diseases or other viral or bacterial co-infections may play roles in the occurrence of complications in this group of patients.

Our study demonstrated that male sex is associated with an increased risk of developing AKI. This finding supports previous studies, which also show that males are more likely to develop AKI [14, 33, 34]. A previous study has also demonstrated that endoplasmic reticulum stress plays a role in the development of AKI in both humans and animal models and that the kidneys of males are more susceptible to such endoplasmic reticulum stress [35]. Furthermore, testosterone has fibrotic and apoptotic properties that can increase the production of TNF-α, in turn triggering the inflammatory process leading to AKI. In our study, a history of diabetes mellitus was also shown to increase the risk of developing AKI. A previous report has also demonstrated similar findings [33]. Persistent hyperglycemia causes microvasculopathy and interstitial inflammation, leading to renal ischemia-reperfusion injury.

Dengue patients with a history of hepatitis B infection are more likely to develop AKI. Hepatitis B may lead to glomerulonephritis due to the deposition of HBsAg and HBeAg, which in turn result in hematuria, proteinuria, and reduced GFR. However, history of hepatitis B infection was not associated with AKI in current study. Conversely, obesity was found to be an associated factor for the development of AKI in our study. An earlier investigation has reported that obesity is a risk factor for AKI, as explained by alterations of renal hemodynamics, which increase the susceptibility to AKI [36]. Additionally, increased production of inflammatory cytokines from adipose tissue in response to acute illness plays a role in the development of AKI. Furthermore, we established that patients with severe dengue are more likely to develop AKI, and this finding is consistent with those in previous studies [8, 34]. Patients with coexisting bacterial infections also tended to develop AKI in our study. These observations are comparable to other studies showing that coexisting bacterial infection or sepsis is a risk factor in developing AKI [16, 37]. The pathogenesis of AKI in dengue with concurrent bacterial infection is related to the cytotoxic effects of inflammation and impaired microcirculation, which may be worsened by the indirect nephrotoxic action of certain antibiotics [38]. Predictive values synthesized from current multivariate regression model are specific for the prevalence of AKI in this study and may change if settings with different prevalence were assessed.

In contrast to all patients in the non-AKI group who had completely recovered at the time of discharge, death occurred in 9 (12.7%) patients in AKI group. All death cases in our study had multi-organ failure, and they were categorized as DHF grade IV, severe dengue, and AKI stage 3. Similar to our findings, a previous study showed that all death cases had occurred in the AKI group [34]. Other studies demonstrated varying mortality rates in dengue patients with AKI, ranging from 0.9% to as high as 60%, the majority of which belonged to the AKI group [9, 15, 16]. These studies reported that mortality resulted from multi-organ failure, severe sepsis, shock, and renal complications. Severe dengue and AKI have been demonstrated to be mortality indicators in dengue patients [37]. In our study, 12.7% (9/71) of patients received vasopressors during hospitalization, all of whom had died. The use of vasopressors has been shown to be associated with the development of AKI [14].

In the current study, of all patients with AKI, 14.1% (10/71) required dialysis, which is higher than the proportion of patients with dengue-associated AKI requiring dialysis reported in two other studies as “only” 1.2% [9] and 7.1% [6]. However, many studies have reported no requirement of RRT in any patient with AKI [25, 33].

Recovery of renal function, defined as either the absence of AKI criteria or discontinuation of RRT following dialysis-requiring AKI [39], was reported in several studies [8, 17], although persistent renal insufficiency has been previously shown to occur in 45.3–48.4% of patients with dengue-associated AKI [33, 40]. Furthermore, dengue patients who have been diagnosed with an episode of AKI are more likely to develop end-stage renal disease (ESRD). After multiple episodes of AKI, nephron loss triggers glomerular hypertrophy in the remaining nephrons, which is worsened by intrarenal hypertension and hyperfiltration [41]. In our study, patients with AKI also showed a longer duration of hospitalization, similar to that reported in other studies [7, 34].

The present study has some limitations. First, we relied only on the serum creatinine level to stratify AKI. Second, we used serum creatinine levels on admission as baseline, which were difficult to determine in some patients. Third, because this was a retrospective study, all of the investigations depended on clinicians’ decision. Fourth, we studied hospitalized adult DVI patients; hence the results cannot be generalized to the whole population of DVI cases. However, our study is strengthened by a large patient population and the comprehensive description of AKI among dengue patients.

## Conclusions

The prevalence of AKI among hospitalized dengue-infected adults in our study was 4.8%. We found that older age, male sex, a history of diabetes mellitus, obesity, severe dengue, and the presence of coexisting bacterial infections were associated factors for developing AKI. Patients with dengue-associated AKI also had a higher mortality compared with that of patients without AKI. Our findings highlight the need for clinicians’ awareness of AKI in DVI, particularly regarding patients with factors associated with a greater risk of developing AKI. The evaluation of clinical and laboratory characteristics of dengue patients will help clinicians initiate timely and appropriate therapy for dengue-associated AKI.
